# Parents’ Multiperspective Views and Misinformation About COVID-19 Mitigation Measures During Tennessee School Board Meetings: Qualitative Content Analysis Using YouTube

**DOI:** 10.2196/75691

**Published:** 2026-04-20

**Authors:** Olufunto A Olusanya, Brianna M White, Brenda Amuchi, Chad Melton, Arash Shaban-Nejad

**Affiliations:** 1 UTHSC-ORNL Center for Biomedical Informatics, Department of Pediatrics, College of Medicine, University of Tennessee Health Science Center, 50 North Dunlap Street, 492R, Memphis, TN, 38103, United States, 1 901 287 5834; 2 Boston University School of Public Health, Boston, MA, United States

**Keywords:** misinformation, mask mandate, vaccines, schools, in-person learning, COVID-19 pandemic

## Abstract

**Background:**

In fall 2021, Tennessee school districts faced heightened debates over COVID-19 mitigation amid rising cases, limited vaccination availability, and widespread misinformation. School board meetings (SBMs) served as pivotal decision-making forums influencing district policies. This study investigated perceptions and misinformation regarding the COVID-19 mask mandate at SBMs held within 6 of Tennessee’s largest school districts. With widespread debate over pandemic measures, including mask use in schools, understanding community sentiments is crucial for guiding public health policies.

**Objective:**

This study aimed to investigate the viewpoints of parents or caregivers and teachers regarding COVID-19 safety protocols, particularly the mask mandate, and to identify the misinformation circulating within SBMs.

**Methods:**

Participants’ commentaries were extracted from 6 SBM recordings that were publicly uploaded to YouTube from August through September 2021. The data were examined qualitatively to capture themes related to concerns, support, and misinformation. Inductive thematic analysis was conducted using transcripts generated via Microsoft Azure speech-to-text and manually verified.

**Results:**

Many parents or caregivers gave personal accounts of how the pandemic had impacted them, their children, and their communities, describing significant comorbidities, adverse psychosocial impacts, mental health disorders, learning difficulties, and worsening socioeconomic and educational disparities. Six thematic domains emerged: (1) perceived effects of the COVID-19 pandemic on children, teachers, and parents or caregivers, including psychosocial distress, learning disruptions, and burnout; (2) perceived effects of mask mandates on children, particularly concerns regarding physical health and psychosocial well-being; (3) perceived government overreach and legal objections to COVID-19 mitigation mandates; (4) tensions between personal liberty, religious beliefs, and collective responsibility in masking decisions; (5) circulation of misinformation and conflicting guidance regarding mask safety and effectiveness; and (6) institutional strain, social tensions, and hostility directed toward school officials alongside educator burnout.

**Conclusions:**

Perspectives on COVID-19 mitigation varied widely across meeting participants, highlighting the need for health officials and policymakers to engage in proactive health promotion strategies. Strengthening public health communication, misinformation mitigation, and institutional support for teachers will be essential to ensuring safe and effective learning environments during future public health crises.

## Introduction

The COVID-19 pandemic, caused by SARS-CoV-2, has significantly impacted public health, education, and society. Emerging in late 2019 in Wuhan, China [1], it continues to negatively affect health care delivery, community well-being, and social equity. By 2023, the United States had recorded more than 104 million COVID-19 cases and 1.1 million deaths [2], underscoring a major public health crisis that strained hospitals, health care workers, and public health infrastructure [3-6]. The pandemic led to school closures and a transition to online and hybrid learning, exacerbating disparities in digital access, educational settings, and academic support [7]. Students from racial and ethnic minorities, low-income households, and with limited internet faced barriers to participation, academic progress, and mental health. Overall, the pandemic worsened long-standing racial and ethnic disparities across the continuum of health care, from testing and vaccination to hospitalizations and mortality, mirroring educational inequities [8,9].

During the COVID-19 pandemic, widespread school closures were implemented across the United States to slow transmission as infection rates increased. As schools reopened through phased or hybrid models, school districts encountered barriers to implementing evidence-based mitigation measures, such as mask use, social distancing, and hand hygiene, due to significant opposition from some parents or caregivers [10-12], despite strong public health recommendations. Before the Pfizer-BioNTech COVID-19 vaccine was authorized for children ages 5‐11 years in October 2021, experts emphasized the importance of maintaining preventive measures [13,14]. By the 2021‐2022 academic year, 18 states and the District of Columbia had mask mandates. However, by May 2022, all states except Hawaii had lifted these requirements, despite evidence that such measures reduced infection and mortality rates [15].

Tennessee, the 15th most populous US state in the southeastern United States, faced COVID-19 unique challenges [16]. In fall 2021, school board meetings (SBMs) were held statewide to disseminate safety information and support in-person learning. These meetings also served as public forums where parents or caregivers could express support or opposition to the mask mandates [17]. Many SBM recordings were uploaded to YouTube, a social networking platform widely known for accessible health education [18,19]. Unlike typical YouTube content, these recordings document civic proceedings without being algorithmically promoted. However, the lack of content oversight, combined with the widespread dissemination of misleading information and conspiracy theories, posed a significant public health challenge [20]. This misinformation, amplified by politicization and antiscience narratives, contributed to mistrust of public health institutions and hindered acceptance of COVID-19 mitigation measures [21]. Unlike popular YouTube health content, SBM recordings tend to reach smaller, local audiences and may not engage the broader public.

Despite rising COVID-19 cases, Tennessee lifted its mask mandate in April 2021 [22], and by July 2022, only 56.54% of residents were fully vaccinated [23]. While COVID-19 mandates have ended, misinformation, mistrust, and polarized health communication persist in debates about other pathogens. Thus, the implications extend beyond COVID-19 specifically. This study was conducted in fall 2021 amid active debates about COVID-19 mitigation policies and limited pediatric vaccination. Our study identifies overarching best-practice themes and offers actionable recommendations to improve preparedness and response in K-12 educational settings during future public health crises. We anticipate that these findings will inform health promotion, policymaking, and program implementation.

While these trends demonstrate resistance to public health recommendations, few studies have qualitatively examined the social and contextual factors influencing public beliefs, behaviors, and hesitance toward mitigation measures and vaccination. This research fills that gap by exploring parents’ and caregivers’ perceptions of the mask mandate and other public health interventions through an analysis of Tennessee’s SBM recordings uploaded to YouTube.

## Methods

### Overview

YouTube provides publicly accessible web-based data used in this study [24]. SBMs, the unit of analysis for the study, were held in person across 6 of Tennessee’s largest counties and were subsequently uploaded to YouTube. In the fall of 2021, we examined YouTube recordings of SBMs conducted between August and September 2021 (ie, before school districts reopened for the 2021‐2022 school year) to gain insight into parent or caregiver perceptions of the universal mask mandate. Fall 2021 was selected as the analytical time point because it coincided with the start of the academic year after statewide COVID-19 mandates were lifted in Tennessee. During this time, school districts were actively deliberating mitigation strategies for the upcoming school year, making it a critical window for examining public testimony and policy debate. While additional time points could provide longitudinal insight, this study was intentionally designed to capture discourse during and immediately after this specific policy transition period. A qualitative analysis was conducted on the commentaries and testimonials delivered during these in-person meetings.

### Study Area and Population

We used a purposive sampling strategy to select 6 large counties based on population sizes using data from the 2021 American Community Survey. Recordings for the SBM were selected based on the following eligibility criteria: (1) SBMs that were held within 6 of Tennessee’s largest counties, sorted from largest to smallest: Shelby, Davidson, Knox, Hamilton, Williamson, and Montgomery, and (2) SBMs that were officially uploaded on the YouTube home pages of school districts. We acknowledge that speakers at SBMs may only represent a self-selected subset of community members; however, because SBMs serve as formal policymaking forums where decisions are directly shaped by public testimony, the views expressed remain central to understanding the local public health decision context.

### Data Collection and Management

The framework that guided our data collection and storage was based on the following stages: (1) extraction and download of video recordings, (2) transcription of video recordings, (3) dual independent review of video recordings, and (4) data storage within a password-protected, Health Insurance Portability and Accountability Act (HIPAA)–compliant drive [25]. Each SBM recording was downloaded directly from each county’s school district YouTube home page to reflect unedited, accurate content. The 6 downloaded video recordings were converted to audio files for transcription. Using Microsoft Azure’s speech-to-text software, audio files were converted to verbatim written transcripts, which were then subsequently deidentified to protect the confidentiality and privacy of SBM attendees. Transcripts were manually reviewed and corrected by members of the research team to ensure accuracy and data quality. Methodological rigor and trustworthiness were supported for this study through the following strategies: (1) credibility, confirmability, and accuracy were enhanced by having 2 independent research teams review all transcripts and conduct data analysis; (2) data saturation was determined once all relevant themes had been identified and no new information emerged [26]; (3) the use of SBM speakers’ verbatim quotes throughout the analytic process to ensure accurate representation of their perspectives; and (4) comparing accounts across a diverse range of speakers strengthened the dependability and completeness of the findings by ensuring that multiple viewpoints were captured [27].

### Ethical Considerations

Growing evidence highlights ethical concerns associated with conducting research using data from social media websites [28]. Accordingly, several ethical considerations were observed in this study to minimize concerns and ensure speakers’ privacy and confidentiality. YouTube video and audio files were uploaded and stored in a password-protected, HIPAA-compliant data storage drive accessible only to research team members. Participants’ personal information (ie, names and demographic characteristics) was deidentified to safeguard confidentiality. Only SBMs publicly uploaded to YouTube and readily accessible to any internet user were used in our research. In addition, speakers’ quotes were reported verbatim in our Results section. Quotations were selected to reduce identifiability by removing personal details and focusing on thematic content. The University of Tennessee Health Science Center Institutional Review Board reviewed the study and determined that it met criteria for exemption from full review (23‐09294-NHSR) [29]. Because the study analyzed publicly available online content and did not involve direct interaction with human participants, informed consent was not required. Participants did not receive compensation for their contributions, as the data analyzed were publicly available recordings of school board meetings.

### Analytical Approach

#### Qualitative Analysis

Using the traditional qualitative method, our study was guided by thematic and inductive analytic approaches [30]. Transcript files were exported into the qualitative software program ATLAS.ti (version 9.0; Scientific Software Development GmbH) for thematic analysis [31]. ATLAS.ti’s artificial intelligence–assisted functions were not used; all coding was conducted manually by the research team. Before analysis, transcribed data were repeatedly read and reviewed in their entirety to ensure an in-depth understanding of the data. Our team was divided into 2 groups, which independently conducted detailed coding of the transcripts. The coding process entailed systematically searching, identifying, collating, categorizing, reviewing, and reporting emerging patterns (themes) embedded throughout the transcript data [30]. Thereafter, coding discrepancies were resolved through consensus within the research team. Codes were subsequently condensed and recategorized to represent broad themes. A detailed codebook and code definitions were developed to elicit essential details from the transcripts. Codes were reassessed, compared, and regrouped into final thematic classifications to ensure consistency. This analytic approach has been previously adopted in another study that qualitatively assessed an online data source [32].

#### Identification of Misinformation

For this study, we defined misinformation as statements that are presented as facts but contradict public health guidance in effect during August-September 2021, evidence-based public health guidance from the Centers for Disease Control and Prevention, American Academy of Pediatrics, and World Health Organization. During coding, 2 researchers (OO and BW) independently flagged such statements and resolved disagreements through discussion, referring to these sources as needed. Value-based opinions were not coded as misinformation unless they contained empirically false factual claims.

## Results

### Overview

Speakers at the SBMs included parents or caregivers, health care professionals, and local government officials. The number of speakers at the SBM includes Davidson (n=32), Knox (n=28), Williamson (n=27), Montgomery (n=25), Hamilton (n=17), and Shelby (n=16). Many parents or caregivers gave personal accounts of how the pandemic had impacted them, their children, and their communities. Six major domains emerged from thematic analysis, summarized in Table 1 with representative participant quotations.

**Table 1.** Summary of key findings and representative quotes from Tennessee school board meetings (August-September 2021).

Domain	Key findings	Representative quotes
Perceived effects of the COVID-19 pandemic on children, teachers, and parents or caregivers	Participants described wide-ranging psychosocial and educational disruptions. Parents reported mental health issues (anxiety and depression), and teachers voiced concerns about safety, staffing shortages, and burnout.	“It’s causing an increased sense of uncertainty, apathy, and anxiety.” “Many of them were very anxious and depressed…one of our students confessed he was thinking about taking his life.” “Will parents be forced to leave work to watch their kids because their schools don’t have sufficient staffing?”
Perceived effects of the mask mandate on children, teachers, and parents or caregivers	Parents frequently opposed mask mandates, citing physical discomfort, respiratory problems, and learning loss. Emotional effects included frustration and fear of stigmatization among children.	“My son has a very difficult time breathing while wearing a mask.” “Do you know what sort of psychological damage we do to children by forcing them to cover their faces?” “It is not healthy for a child to think that they are a disease vector.”
Perceived government overreach and legal objections to COVID-19 mandates	Participants debated the legality and ethics of mask mandates. Some argued school boards lacked authority; others viewed mandates as ethical acts for collective safety.	“This board does not have the legal right to issue a mask mandate.” “Making choices for the collective good is exactly what I want to model.” “You have the responsibility to take care of the school and the current health environment.”
Perceptions of liberty and religious freedom as core to American identity	Many framed opposition around parental rights and religious beliefs, equating mandates with loss of freedom. Others stressed balancing personal liberty with social responsibility and community health.	“You are not God ... To impose your will over free individuals is un-American.” “Our beliefs guarantee my freedom, and my children’s, to breathe oxygen.” “To be a part of any organized, healthy community requires some abandonment of personal freedoms.”
Misinformation and conflicting guidance on mask use	Misinformation and pseudoscientific claims circulated widely. Some cited unverified medical opinions asserting masks were harmful or ineffective, illustrating the influence of misinformation during the pandemic.	“There is no evidence that masks decrease the spread of COVID-19.” “Masks cause issues for children ... microbes are inhaled deeply into the lungs.” “People engaging in cosmetic theater thinking they are making a difference.”
Institutional strain, conflict, and social tensions in school-based COVID-19 mitigation	Meetings revealed polarization and hostility toward school officials. Personal attacks and threats were common. Educators described exhaustion, lack of support, and inconsistent policy guidance.	“You have decided that our kids should go to school every day wearing muzzles like rabid dogs.” “If you vote for this, we will vote you out.” “Deaths of teachers—no change.”

### Major Themes and Findings

#### Perceived Effects of the COVID-19 Pandemic on Children, Teachers, and Parents or Caregivers

##### Concerns of the COVID-19 Pandemic on Health and Health Care Systems

The COVID-19 pandemic caused significant morbidity or mortality and placed an enormous burden on health personnel. One health care professional shared firsthand accounts of the severity of COVID-19 within clinical settings and expressed concern for decreasing COVID-19 preventive measures:

*... kids are getting sick ... Pediatric ICUs and ERs across the country, across the South are being stretched to capacity ...*

Another echoed this concern:

*Our pediatric hospitals are already full of RSV and parainfluenza. There will not be staff to care for the children when they get sick.*

One teacher shared:

*Once I got sick ... I was in the hospital twice. I’ve been diagnosed with ... multiple life-threatening illnesses as a consequence of COVID, including several heart conditions ...*

Some conveyed their frustrations at others’ disregard for COVID-19 safety measures:

*It is like you were throwing a large Chicken Pox party with our children—only the stakes are incredibly higher. How can you not prioritize the life of a child?*

##### Concerns About the COVID-19 Pandemic on Psychosocial Capabilities, Staff Shortages, and Student Learning

Some SBM speakers expressed that significant psychosocial impact and adverse health effects had created devastating effects. While some expressed their support for COVID-19 mitigation measures, many conveyed feelings of pandemic fatigue and burnout. Some parents or caregivers were concerned about school staffing shortages:

*Will we need to shut down the schools, and will parents be forced to leave work to watch their kids because their schools don’t have sufficient staffing ...?*

Referring to psychosocial impacts and increased frequency of mental health disorders among students, SBM speakers shared:

*... the threat of reinstating restrictions is very upsetting to students. It’s causing an increased sense of uncertainty, apathy, and anxiety ...*

and

*... many of them were very anxious and depressed ... It has affected their mental health so poorly one of our students ... confessed that he was thinking about taking his life ...*

Significant disruptions to learning and in-school activities were discussed. One teacher articulated concerns about non-English–speaking students:

*... for students with exceptionalities and English language learners, that is still a big deficit.*

Moreover, some parents or caregivers expressed the following criticism:

*... contact tracing needs to stop. The kids that went home, they sat there and stared at the walls for two weeks and then went back to school ...*

and

*... last year he was contact traced three times ... so, he missed eight weeks out of 18. The weeks that he was home, he did literally nothing ...*

### Perceived Effects of the Mask Mandate on Children, Teachers, and Parents or Caregivers

#### Concerns About the Use of Masks on Children’s Health

The sentiments on the mask mandate were negative, with several parents or caregivers opposed to their implementation in schools. One parent shared:

*My son has a very difficult time breathing while wearing a mask. ... he has had croup six times, RSV twice, and pneumonia, which has compromised his lungs and made wearing a mask for long periods extremely difficult.*

Other parents or caregivers echoed this sentiment:

*My four children went with a mask ... They hated it daily. We had headaches, stomachaches, tears about not wanting to go to school.*

Another shared:

*... I have two vaccine-injured children ... still I would never put them in a mask because their brain needs oxygen to grow ...*

#### Concerns Regarding the Use of Masks on Children’s Psychosocial Capabilities

While discussing the emotional and adverse impact of the masks, one parent shared:

*Now, do any of you know what sort of psychological damage we do to children by forcing them to cover their faces? ... depriving them of the ability to see each other’s faces ... Have you wondered about the health effects of forcing kids to breathe through sweat, spit, and dirt-soaked rags every single day?*

Moreover, some parents or caregivers expressed their fears about masks leading to learning loss:

*But what we’re not talking about is ... the learning loss and the behavioral issues that have popped up from wearing these masks.*

Similarly, one parent noted the undue social burden on children who were sometimes viewed as a public threat for not wearing masks:

*... consider the profound mental health conditions that you are potentially instilling in your children. It is not healthy for a child to think that they are a disease vector, going around killing their grandparents and their neighbors.*

### Perceived Government Overreach and Legal Objections to COVID-19 Mandates

#### Distrust of Laws Regarding the Implementation of Masks

During the SBM, some parents or caregivers referred to the state government’s role, or perceived lack of it, in implementing COVID-19 safety measures, arguing that the proposed mask mandate was unlawful. One parent asserted:

*Somebody brought up the governor. ... he’s not an interpreter of the law. We have an interpreter of the law who told us that this board does not have the legal right to issue a mask mandate.*

Another added:

*Masks and vaccines are emergency use authorizations. They are not to be mandated under federal law. The law is on our side, the science is on our side.*

One parent threatened to take legal action, “If this board exceeds their legal authority and votes to mask our kids, how many people here will sign on to a class action …?” However, others acknowledged that school districts did have the authority to impose mask requirements, noting, “The governor said the way laws are set up in Tennessee, school districts have the authority to institute mask requirements.”

#### Perceptions of Collective Responsibility Versus Individual Choice in Masking Decisions

While some parents or caregivers opposed the implementation of school mask mandates, others viewed them as a collective effort and a selfless choice. One parent shared, “How do I explain to my first-grade daughter that she needs to wear a mask *...* but other kids don’t have to wear masks because their parents want them to have a normal experience, and personal choice is more important than the health of a community.” A teacher emphasized the importance of modeling community-oriented behavior, stating, “*...* I try to facilitate with my kids *...* making choices and taking actions for the collective good is exactly what I want to model.” Several individuals urged school board members to prioritize student safety: “As a county school board, you have the responsibility to take care of the school and the current health environment.” However, some parents or caregivers interpreted these expectations differently, arguing, “It’s also a violation of ethics for this board to vote in a partisan manner and not represent their constituents *...*”

### Perceptions of Liberty and Religious Freedom as Core to American Identity

#### Perceived Parental Right to Decide the Child’s Masking Behavior

Some parents or caregivers emphasized their right to determine what was best for their children, expressing concern that mask requirements threatened their personal freedoms. One parent stated, “You are not God *...* To impose your will over the power of free individuals is not only morally reprehensible, it’s un-American.” Another parent added, “*...* the constitution and the Declaration of Independence, the Bill of Rights, and the Federalist Papers, and also the Bible *...* these guarantee my freedom and yours, and our children to breathe oxygen.” One parent explained that the reluctance to wear masks was motivated by their religious beliefs: “Here’s the email that I got when I secured my son’s religious exemption that he would not have to wear a mask anymore because it goes against our beliefs.”

Nevertheless, several parents or caregivers supported universal masking: “To be a part of any organized, healthy community requires some abandonment of personal freedoms.” Another parent criticized what was perceived as misplaced priorities on dress code policies, “Or should I tell her (daughter) that she has to wear certain clothes—but don’t worry, you don’t have to wear masks because you’re not responsible if you give your friends a virus ...”

#### Prioritizing Students’ Right to In-Person Learning or Education and Safety

Discussions also centered on barriers to implementing a typical back-to-school experience. In contrast to arguments focused on parental rights and personal freedoms, several parents or caregivers emphasized students’ rights to safety and uninterrupted education. One parent argued: “This decision is not about parents’ rights—this is about students’ rights to safe learning environments *...*.” Another highlighted concerns about harassment and harmful rhetoric:

*It is not okay for students to pull off the masks of other students who choose the right to wear masks. It is not okay for a teacher to make fun of students that choose to wear masks. It is not okay to compare wearing a mask to child enslavement or the Holocaust.*

For these parents or caregivers, returning to school with a mask mandate was seen as one of the most effective strategies to ensure a safe, stable, and consistent in-person learning environment.

### Misinformation and Conflicting Guidance on Mask Use

The COVID-19 pandemic appeared to exacerbate the infodemic, with misinformation amplified through politicized debates surrounding mask mandates, vaccinations, and other mitigation measures. While the majority of health care providers advocated for mask use, a minority of health care providers offered contradictory guidance. One physician stated:

*I’m a physician—I believe in statistics—I follow the science. There is no evidence that masks decrease the spread of COVID-19. Masks cause issues for children ...*

While a parent cited recommendations received from their health care provider, claiming that masking could worsen illness severity:

*Dr. [Insert name] states that asymptomatic or mild cases of COVID become more severe when the infected is masked .... The oxygen-lowering effects of masks cause these microbes to be inhaled deeply into the lungs, where respiratory disease far worse than COVID can result.*

Some parents or caregivers expressed skepticism toward public health measures more broadly, with one noting, “I feel bad for all these people engaging in cosmetic theater thinking that they are making a difference against COVID.” Others highlighted knowledge gaps and long-term safety concerns, stating, “*...* we don’t know enough about it, and I’m not willing to risk the long-term health of my child breathing in from a mask that we are meant to exhale.”

### Institutional Strain, Conflict, and Social Tensions in School-Based COVID-19 Mitigation

#### Personal Attacks on School Officials

Some parents or caregivers expressed appreciation for the schools’ efforts to implement COVID-19 mitigation measures. However, others directed sharp criticism at school board members, with one stating, “You and the school board have decided that our kids should go to school every day wearing muzzles like rabid dogs.” In some cases, opposition escalated into explicit threats: “If you vote for this *...* we will vote you out—if you own a business, we will boycott your business *...*, you become a domestic enemy, and we will come for you politically and financially.” These attacks were occasionally followed by loud clapping, whistling, and cheering from some attendees.

#### Teacher Burnout and Lack of Institutional Support

Teachers attending the SBM also expressed fatigue and burnout. Many highlighted the pressures of navigating virtual and in-person learning while facing limited support, including insufficient sick leave, minimal pay increases, and low staffing levels. Moreover, conflicting health guidelines and policies from school districts further contributed to a sense that teachers’ health was not a priority. “Last year, when my spouse was showing signs of COVID, I was told to come in because I was vaccinated…” Another added, “Now the data shows different and deadly strains, yet no change *...*. Deaths of teachers—no change.”

#### Privacy Concerns and Social Shaming

During the SBM, the COVID-19 vaccination had been approved for children aged 12 years and older. Accordingly, some parents or caregivers expressed concerns about disclosing students’ COVID-19 vaccination status. A few parents or caregivers noted that teachers publicly asked students to share this information, stating:

*... there have been middle school teachers who have asked the students ... to raise their hand if they’ve been vaccinated. I have heard administrators at assemblies this week who have praised and thanked children who are wearing masks.*

One parent criticized this practice as social shaming:

*They are asking minor students to share protected health information in a public arena, which violates their privacy. Calling them out in front of others is social shaming.*

Echoing this concern, another parent remarked:

*... we’re making things even more divisive for our children ... this student is wearing a mask, and the student isn’t ...*

## Discussion

### Principal Findings

Taken together, the 6 thematic domains highlight recurring patterns in SBM discourse, including contested authority over decisions related to children’s health, the use of identity- and value-driven framing that facilitated the spread of misinformation, and increasing institutional strain as they navigated their role in public health decision-making. These patterns reflect broader challenges described in prior works on public trust, health communication, and the governance of public health interventions in educational and community settings [21,33-36].

Public schools are a central site where decisions about child health, education, and public welfare intersect. In fall 2021, SBMs across Tennessee became a key forum for considering COVID-19 mitigation strategies, including mask mandate, amid heightened concern, uncertainty, and debate. To examine how authority over child health decisions was negotiated in these settings, we examined comments and testimonials from SBM recordings uploaded to YouTube by school districts. Our analyses were conducted using qualitative thematic analysis of extracted data. Our findings revealed a significant divide among speakers. A subset of parents or caregivers strongly supported the mask mandate, emphasizing the importance of ensuring students’ safety and maintaining the continuity of in-person learning during the pandemic, while others raised concerns grounded in personal values, perceived rights, and skepticism toward public health guidance. These findings underscore the complexity of balancing public health measures with community values and the challenges of addressing misinformation that may shape public opinion. These themes resonate with the principles of precision health [37], which position community perceptions, local sociopolitical environments, and real-world behavioral contexts as integral components of effective prevention.

However, some parents or guardians opposed local mask mandates in educational settings, citing violations of parental rights and infringement on personal liberties. Several SBM speakers described significant fears and concerns about the impacts of the COVID-19 pandemic, its mitigation measures, and other unfavorable sequelae (eg, disruptions to learning and mental health impacts). According to SBM commentaries, the COVID-19 pandemic and public health prevention measures had negatively affected students, parents or caregivers, and teachers. As in other reports, these SBM open forum sessions were occasionally characterized by highly politicized debates among some attendees who expressed negative sentiments, often expressed by other influential people [33] toward COVID-19–related policies and mitigation measures [17]. Scientific misinformation and conspiracy theories were circulated by some parents or caregivers and a few health care professionals who spoke outside their areas of expertise. During these meetings, debates emerged over whether vaccine and mask mandates were constitutional or infringed on personal liberty. In contrast, parents or caregivers who supported mask mandates emphasized students’ rights to a safe learning environment and access to quality education. Religious objections primarily centered on perceived encroachments on parental authority rather than doctrinal prohibitions, which is consistent with available literature, indicating that religious resistance to masking reflects broader ideological and identity-based concerns rather than theological mandates [34,38-41].

The proliferation of misinformation posed a significant challenge to disease prevention and containment measures during the COVID-19 pandemic. The widespread availability of unverified user-generated content on social media platforms, combined with unrestricted discourse that amplified incomplete or false narratives, undermined vaccine acceptance and deterred adherence to COVID-19 mitigation measures [35,36]. Likewise, parental fears and concerns about vaccine side effects, poor health literacy and information-seeking behavior, and lack of knowledge have eroded public trust in vaccines and public institutions [42]. Using four overarching themes, we offer recommendations to facilitate decision-making for the K-12 school system and bolster health promotion preparedness strategies and response for future outbreaks or pandemics: (1) implement evidence-based safety measures; (2) address misinformation using culturally appropriate, trustworthy, clear, and timely messages; (3) prioritize in-person learning and student support services; and (4) facilitate continued collaboration and engagement of relevant stakeholders. Our findings can help health officials and policymakers design policies to support school decision-making in districts with similar sociopolitical and demographic contexts.

Themes from the SBMs, particularly concerns about student safety, teacher burnout, and confusion over public health guidance, suggest that maintaining in-person learning during outbreaks requires locally trusted, clearly communicated, and context-specific safety practices. To reduce transmission during periods of high COVID-19 community spread, or during future outbreaks, school districts should collaborate with public health authorities and health care providers to promote vaccinations, booster doses, and routine childhood immunizations, including catch-up doses for those who missed them. SBM speakers frequently described uncertainty and conflicting guidance. To address this, school districts should collaborate with local public health authorities to ensure that mitigation strategies, whether masking during periods of high transmission, improving ventilation, or offering accessible testing options, are communicated in a consistent, evidence-informed manner that reflects evolving guidance. These measures align with stakeholder concerns raised in SBMs and help reduce confusion among parents, caregivers, and teachers. Continued government-supported programs are essential to ensure equitable access to vaccines, therapeutics, and testing, particularly for high-risk populations and underserved communities.

The World Health Organization refers to the surge of misinformation during the COVID-19 pandemic as an “infodemic,” highlighting the rapid spread of false or misleading information that undermines public health efforts [43]. Misinformation poses a serious threat to the public because it prolongs outbreaks and pandemics when individuals are confused and distrustful about preventive measures. Evidence-based strategies are recommended to tackle the spread of false online information, including providing training to recognize fake news, dispel pseudoscientific health beliefs and practices, and increase the visibility of accurate, science-based information across media platforms [44]. Strengthening automated systems and reliable algorithms to identify and flag false online content is crucial [45]. In addition, targeted public health approaches that include health education and promotion, sensitivity to local social norms, and meaningful community engagement are essential for rebuilding trust and supporting informed decision-making [46,47]. Public health information should be trustworthy, timely, and tailored to key audiences, that is, parents or caregivers, teachers, and students. Addressing misinformation remains essential, particularly as schools continue to navigate COVID-19 alongside other respiratory illnesses such as influenza and respiratory syncytial virus. Current guidance from leading pediatric and public health organizations emphasizes that masks are safe for children, including those with most medical conditions, and are not associated with breathing problems, learning difficulties, or anxiety [13]. Multiple studies continue to demonstrate that high-quality masks reduce the emission and spread of respiratory particles and pathogens, including coronaviruses and influenza viruses [48,49]. Information should be shared in community languages to build trust and reach non-English–speaking parents or caregivers. Surveillance and real-time reporting systems could improve access to updates for all parents or caregivers and support timely health decisions.

In-person learning is essential for tackling educational, racial, social, and nutritional disparities. Moreover, in-person learning could promote students’ well-being and create a safe working environment for teachers. Conversely, school closures and disruptions to in-person learning can be associated with lost future earnings [50,51] and unfavorable economic implications [52] over the life course of affected children. The benefits of keeping schools open are profound because in-person learning facilitates real-time, personalized interactions with teachers or peers; enables healthy, supportive social relationships; and builds students’ identity and sense of self [13]. After COVID-19, teachers have continued to navigate an evolving educational landscape by balancing in-person instruction with support for students recovering from disruptions. To improve future readiness for respiratory illness surges, districts can invest in staffing, provide paid leave after exposure or illness, and streamline contact tracing and illness notification in line with public health guidance [53,54].

Finally, active collaboration and engagement across all stakeholders (ie, the government, researchers, school administrators, and policymakers) remain essential. School authorities can benefit from establishing coalitions that bring together public health experts, mental health specialists, communication experts, health care providers, parents or caregivers, and teachers to offer psychological support to students who continue to face challenges related to grief, academic disruption, social isolation, and ongoing uncertainty. These partnerships can help deliver timely, locally relevant, and age-appropriate information on respiratory illnesses and mitigation practices, addressing the confusion and misinformation that speakers commonly raised during SBMs. SBM speakers frequently described burnout, limited sick leave, and insufficient support. Addressing these concerns, such as by expanding access to mental health resources, ensuring adequate paid sick leave, and promoting work-life balance policies, may strengthen teacher well-being and support school resilience during future public health challenges [55].

### Strengths and Limitations

Several strengths and limitations should be considered alongside this study. One notable strength is the use of publicly available YouTube recordings of SBMs, which provide an accessible and diverse range of parental perspectives on COVID-19 mask mandates. This approach enabled the capture of real-time, spontaneous reactions to pandemic-related policies, which may not have been possible with other research methods. Additionally, the public nature of these forums offered a broad range of opinions, reflecting varying levels of support or opposition to health measures across Tennessee communities. However, this also presents a limitation, as the data were sourced from a social media platform, raising concerns about potential risks to participant privacy and confidentiality. Online forums make it hard to ensure users understand data sharing, possibly affecting their disclosures. The lack of face-to-face interaction limits the ability to ask immediate questions, seek clarifications, and probe, which may reduce data depth. Additionally, the study examined 1 SBM per district, providing a snapshot of community discourse at a given point in time. Moreover, given that public testimony at SBMs is voluntary, speakers may represent individuals with stronger or more polarized opinions, while those with moderate views may be less inclined to speak publicly. Therefore, the perspectives captured may not fully reflect the distribution of attitudes within the broader community.

Data were collected about a year into the pandemic, around the initial vaccine rollout, which may have influenced attitudes, concerns, and debates. Public perceptions then were shaped by evolving science, policy changes, and vaccine access and acceptance. Finally, the results’ transferability is limited. Tennessee’s political climate, demographic makeup, community values, and local mitigation policies may differ greatly from those in other states or regions. Therefore, while themes provide insights into broader national trends, caution is needed when applying findings elsewhere without considering local sociopolitical and cultural factors. Despite these issues, the study offers valuable insights into public discussions about school mask mandates and highlights areas for improving health messaging and policies. Findings may be particularly relevant to regions characterized by political polarization, strong local control over school governance, or communities where public health measures intersect with ideological debates.

### Conclusions

Future research could examine how perspectives expressed in SBMs change over time, particularly as public health guidance, vaccine availability, and community transmission patterns evolve. Longitudinal studies may reveal shifts in parental concerns, trust in health authorities, and attitudes toward mitigation during different pandemic phases. Expanding research beyond Tennessee would assess if themes were consistent across regions. Comparative analyses could identify regional differences in misinformation, policy debates, and stakeholder engagement. Further research could examine how SBM discussions affect local policies, school implementation of health recommendations, and community adherence to mitigation strategies.

This study provides real-time insight into how parents or caregivers, teachers, and community members articulated concerns about COVID-19 mitigation in public school settings during a period of active uncertainty. The themes explored illustrate how misinformation, political polarization, and competing moral frameworks shaped local decision-making. These findings are relevant and useful for preparation against future outbreaks and highlight a need for transparent communication, early engagement with caregivers, and collaboration across stakeholder groups. Recognizing diverse perspectives helps health officials and policymakers develop more effective, inclusive strategies that address public concerns and build trust. A proactive approach to policy and communication is essential to keeping educational systems adaptable, resilient, and able to safeguard health and maintain in-person learning during outbreaks.

## Abbreviations

HIPAA: Health Insurance Portability and Accountability Act
SBM: school board meeting
